# Vulliet’s New Method of Dilating the Uterine Cavity

**Published:** 1886-06

**Authors:** 


### VULLIET'S NEW METHOD OF DILATING THE UTERINE CAVITY.

---

TRANSLATED FROM AN ARTICLE BY BETIX, IN THE NOUVELLES ARCHIVES D'OBSTETRIQUE ET DE GYNECOLOGIE OF JAN. 25, 1886, BY VIRGIL O. HARDON, M. D., ATLANTA, GEORGIA.

---

At the October meeting of the *Societe Medicale de Geneve*, Professor Vulliet presented a communication concerning his process of progressive and continuous dilatation of the uterine cavity, a process which he has introduced in his private practice and of which he has made very frequent use in the gynæcological service of the Maternité during the months of July, August, September and October, 1885. Having had the privilege of serving as assistant to that service during that time, I shall endeavor, with the kind consent of Prof. Vulliet, to give some details of the *technique* of this progressive dilatation, and of the results which have been obtained by it.

The object of this procedure is to render visible the internal surface of the uterus throughout its whole extent and to permit the inspection of the uterine cavity in the smallest folds of mucous membrane by the naked eye, without the aid of any special optical instrument, the light of the sun or of any ordinary lamp furnishing sufficient illumination. When once the uterine cavity is opened and dilated, it is possible to carry to any part of the wall of the uterus, under direct control of the eye, any instrument, such as a bistoury, curette, caustic or actual cautery, with as much ease and precision as in operations upon external organs.

The manner of dilatation is as follows: The position of the patient is of prime importance. That which is preferable, I may even say indispensable, is that of Bozeman [the writer makes the not infrequent error of ascribing to Bozeman the genu-pectoral position, the credit of which unquestionably belongs to Dr. H. F. Campbell, of Augusta, Ga.], or the genu-pectoral position. The perineum should be forcibly elevated by a Sims' speculum of as large size as possible. In case the pathological process, which necessitates or justifies the dilatation, has not produced more or less patency of the womb, the dilatation should be commenced by inserting any instrument which can be easily introduced, such as a uterine sound or a urethral bougie. The size of the instrument should be gradually increased, choosing in each case such a one as is best adapted to the size and shape of the cervical canal. When a certain degree of dilatation has been obtained by this means, the further procedure is the same as when the canal has been normally or pathologically dilated. It is at this point that the use of iodoformized intra-uterine tampons is to be commenced.

These tampons of cotton vary in size from that of a pea to that of an almond. A double thread is attached to their centre. They are prepared by plunging them into a ten per cent. ethereal solution of iodoform, drying them rapidly by exposure to the air and keeping them in a well-stopped bottle. These tampons are to be carried to the external os by long curved dressing forceps. They are then pushed little by little into the uterine cavity by a stiff metallic rod, having the size and curve of a uterine sound. They should pass completely through the internal os into the uterine cavity. The threads should be tied together into a single knot and passed into the vagina. Thus, at the first sitting, several tampons may be introduced, three, four, or even more, and they should be allowed to remain for twenty-four or forty-eight hours.

At the end of that time the tampons should be removed and, after washing out the uterine cavity, they should be immediately replaced by others, the number being increased at each sitting. The cavity should never remain entirely empty, as thus would be lost all the ground which has been gained.

As a rule, the uterus shows a remarkable tolerance of these tampons. Ordinarily no reaction occurs. Occasionally some pain is produced, but only at the commencement of the dilatation, and it does not last long. Vulliet has noticed in one of his patients a nausea similar to that of pregnancy. After the first sitting there is sometimes a slight rise of temperature for one or two days. I have never seen a chill.

In cases where it is desirable to hasten the first steps of the dilatation, the iodoform tampons may be used for a day or two to secure complete asepsis of the uterine cavity, and may then be replaced by laminaria tents, which should be removed in twenty-four hours and the tampons introduced anew. The laminaria has the advantage of hastening the dilatation and producing a more uniform expansion. But it also has the disadvantage of producing traumatism, and is more painful to the patient.

With the tampons the operator plugs the uterus as a dentist plugs a carious tooth. The cavity is distended little by little, and advantage is taken of the space thus gained to continually increase the number of tampons, until finally the degree of dilatation is such that when the tampons are removed and the air allowed to enter, the whole interior of the uterus is visible, even to the fundus, and the uterine cavity and the vaginal cavity have the appearance of a continuous canal.

Vulliet makes use of this method only when it is necessary or of benefit to the patient. When the introduction of the finger or the curette suffices for the treatment, dilatation is pushed only to the extent necessary for that purpose. He has employed it in a number of cases of cancer of the uterus, in three cases of submucous fibrous polypus, in two large fibro myomata, and in one or two cases of endometritis.

In regard to the time required for complete dilatation, it varies in different patients, according to the condition of the uterus and the amount of resistance of the neck. Usually it takes about fifteen days. When once accomplished, the dilatation may be maintained for a long time. In one case it was kept up for a year. The uterus contracts according as the quantity of cotton is diminished; and when it is increased, the organ expands more rapidly and easily than at first. If the dilated uterus is left perfectly empty, it takes a long time, often many weeks, for it to regain its normal capacity.

Thus it will be seen that continuous uterine dilatation is very practicable. It is true it is often a long procedure, and requires much patience on the part of both surgeon and patient. In many gynæcological cases it is capable of rendering valuable aid, not only as a means of confirming diagnosis, but especially in the application of direct local treatment under the control of the eye, a thing which up to the present time has been very difficult to accomplish. It greatly facilitates local therapeusis in many uterine affections, and will doubtless in the future find more and more frequent use.

